# An Adolescent Boy with Comorbid Anorexia Nervosa and Hashimoto Thyroiditis

**DOI:** 10.4274/jcrpe.2297

**Published:** 2016-03-01

**Authors:** Melis Pehlivantürk Kızılkan, Nuray Kanbur, Sinem Akgül, Ayfer Alikaşifoğlu

**Affiliations:** 1 Hacettepe University Faculty of Medicine, İhsan Doğramacı Children’s Hospital, Clinic of Pediatrics, Division of Adolescent Medicine, Ankara, Turkey; 2 Hacettepe University Faculty of Medicine, İhsan Doğramacı Children’s Hospital, Clinic of Pediatrics, Division of Pediatric Endocrinology, Ankara, Turkey

**Keywords:** Anorexia nervosa, Hashimoto thyroiditis, adolescent, hypothyroidism

## Abstract

Low triiodothyronine syndrome is a physiological adaptation encountered in anorexia nervosa (AN) and generally improves with sufficient weight gain. However, when a primary thyroid pathology accompanies AN, both the evaluation of thyroid hormone levels and the management of the co-morbid disease become more challenging. Hashimoto thyroiditis could complicate the management of AN by causing hyper- or hypothyroidism. AN could also negatively affect the treatment of Hashimoto thyroiditis by altering body weight and metabolic rate, as well as by causing drug non-compliance. We present the case of a 15-year-old boy with comorbid AN restrictive sub-type and Hashimoto thyroiditis. In this case report, we aimed to draw attention to the challenges that could be encountered in the diagnosis, treatment, and follow-up of patients with AN when accompanied by Hashimoto thyroiditis.

**WHAT IS ALREADY KNOWN ON THIS TOPIC?**

Review of the literature for the association of primary thyroid pathologies with eating disorders offers case reports evaluating the deteriorating effect of hyperthyroidism on the psychological and physiological symptoms of anorexia nervosa (AN) such as anxiety, irritability, and fear of losing control over eating due to an increased appetite. In these cases, hyperthyroidism is mostly due to graves’ disease. Also, a recently published case report of a 16-year-old girl with a 4-year history of AN diagnosed with Hashimoto thyroiditis suggests an association between weight and thyroid-stimulating hormone levels.

**WHAT THIS STUDY ADDS?**

Although Hashimoto thyroiditis is accepted as one of the most common autoimmune endocrine disorders, its effect on eating disorders seems to be lacking in the literature. This case of AN with Hashimoto thyroiditis is informative as it highlights the challenges faced during the diagnosis, treatment, and follow-up of Hashimoto thyroiditis in the presence of AN and attracts attention to the effects of hypothyroidism and hyperthyroidism on the patient.

## INTRODUCTION

Anorexia nervosa (AN) is a psychiatric disorder with significant neuroendocrine consequences. In patients with eating disorders (EDs), the hypothalamic-pituitary-thyroid axis is possibly the most studied neuroendocrine pathway. Thyroid function tests of patients with AN resemble the sick euthyroid state which is characterized by low total triiodothyronine (T3) levels, usually normal thyroid-stimulating hormone (TSH) levels, and normal or slightly low thyroxin (T_4_) levels (1,2). This physiological adaptation state is also known as low T_3_ syndrome. Decreased deiodination of T_4_ to T_3_, increased peripheral conversion to reverse T_3_, and decreased thyroidal T_3_ secretion in response to endogenous TSH are the probable causes of this disorder (3,4). T_3_ is a negative measure of the nutritional status, and low T_3_ levels are necessary for the body to protect itself from starvation by limiting resting energy expenditure and conserving energy for vital functions. With nutritional rehabilitation and a sufficient amount of weight gain, T_3_ levels are expected to increase and to attain the normal range (5).

Hashimoto thyroiditis, which can lead to hypothyroidism and sometimes to hyperthyroidism, is a condition which needs to be considered in the differential diagnosis of AN. The laboratory and clinical resemblance of hypothyroidism and AN could complicate the diagnosis and management of both diseases, while a hyperthyroid state could deteriorate the psychological and physiological symptoms of AN.

In this case report, we present these challenges encountered in the diagnosis, treatment, and follow-up of a patient with AN restrictive sub-type accompanied by Hashimoto thyroiditis.

## CASE REPORT

We present a 15-year-old boy who was admitted to our hospital with significant weight loss, malaise, and cold intolerance. Written informed consent for presentation was obtained from the patient and his parents.

The patient weighed 102 kg until four years ago, at which time, with the help of a dietitian, he started to lose weight. However, after losing 2 kg, he discontinued the dietary regime. Two years later, when he weighed 100 kg, he decided to lose weight again because he felt uncomfortable with the way he looked and felt very overweight. Within a year, he lost 20 kg by eating less and playing basketball every day for approximately two hours a day. At that time, he had to quit basketball due to a busy school schedule which led to a fear of gaining weight, causing him to restrict his diet even more. By restricting his daily intake to 500 kcal, he had lost 17.5 kg within the last two months before presenting to our clinic. The patient denied having body image problems but agreed that he had an intense fear of gaining weight. Past medical history was unremarkable except for an appendectomy performed when he was 7 years old. The family history revealed that two of his aunts have Hashimoto thyroiditis.

At admission, the patient’s body weight was 60.7 kg (50-75^th^ percentile). Height was 186 cm (>97^th^ percentile) and body mass index was 17.55 kg/m2 (<3^rd^ percentile). His body temperature was 36.1 °C and respiratory rate was 22/min. His supine blood pressure was 100/60 mmHg and heart rate was 40 bpm. His standing blood pressure was 95/60 mmHg and heart rate was 66 bpm. Cardiac examination was normal except for the bradycardia, and other systems were also normal on his physical examination. Meeting the diagnostic criteria of the fourth edition of Diagnostic and Statistical Manual of Mental Disorders (DSM IV), he was diagnosed with AN-restrictive type and hospitalized due to bradycardia. His laboratory investigations which included complete blood count, liver and kidney function tests, glucose and electrolyte levels, sedimentation rate, cortisol, cholesterol levels, and urinary analysis were all within normal ranges. Thyroid function tests revealed very low TSH levels (0.025 uIU/mL, normal range: 0.27-4.20 uIU/mL), low free T_3_ (fT_3_) levels (2.87 pmol/L, normal range 3.10-6.70 pmol/L), and normal free T_4_ (fT_4_) levels (21.9 pmol/L, normal range: 12.00-22.00 pmol/L). Thyroid peroxidase antibodies and thyroglobulin antibodies were high, while TSH receptor antibodies were negative. With these findings, he was additionally diagnosed with Hashimoto thyroiditis. Thyroid ultrasonography confirmed the diagnosis.

In the inpatient unit, the patient was followed by an interdisciplinary team consisting of a child and adolescent psychiatrist, an adolescent medicine specialist, a pediatric endocrinologist, and a dietitian with special training and experience in adolescent EDs. During his three-week stay, he gained 4 kg. Despite the weight gain and the improvement in his nutritional status, bradycardia continued (40-50 bpm). Echocardiography findings were normal, and Holter monitoring only revealed sinus bradycardia. Thyroid functions were monitored closely without any medical treatment, along with his vitals. Before discharge, while TSH levels were still low (0.018 uIU/mL), fT_3_ levels were thought to be relatively high (3.76 pmol/L) considering his metabolic status. The patient was discharged with a weight of 64.7 kg. At his follow-up visit 2 months later, the boy had gained weight and weighed 75.1 kg. Due to the gradual increase noted in his TSH levels (from 8.91 to 28.84 uIU/mL), levothyroxine treatment was started. At that time, fT_3_ and fT_4_ levels were measured as 7.74 and 4.42 pmol/L, respectively. Two months later, it was learned that he had been using levothyroxine in doses three times higher than the recommended dose. Although monitored closely, due to the drug compliance problems and weight changes with severe body image issues, it was hard to maintain the thyroid levels within a stable course. Stabilization occurred after ten months of therapy when he started using a proper medication schedule and succeeded in preserving his target weight. The course of the thyroid function tests is given in Table 1. Bradycardia also improved with the recovery in thyroid hormone levels.

## DISCUSSION

In the presence of an unexplained weight loss, decrease in appetite, and abnormal eating attitudes, EDs should be considered. However, other medical diseases leading to a similar clinical status such as hyperthyroidism, Addison’s disease, diabetes mellitus, malignancy, inflammatory bowel disease, immunodeficiency, malabsorption, chronic infections, and collagen vascular diseases, should also be included in the differential diagnosis (6). Although concern about the cause of weight loss is helpful in the differentiation of EDs from other diseases, coexistence of a medical disease and an ED complicates the diagnosis and management of both conditions. Within the group of diseases listed above, hypothyroidism is of particular importance as its laboratory findings as well as its clinical findings such as cold intolerance, constipation, hypothermia, and bradycardia highly resemble those of ED’s.

Hashimoto thyroiditis is a chronic autoimmune inflammation of the thyroid gland and it is the most common cause of acquired hypothyroidism in adolescents (7). The hypothesis that autoimmune mechanisms are involved in the pathogenesis of EDs has become more acceptable in recent years, supported by the results of several studies indicating that autoimmune diseases occur in higher ratios in ED cases (8,9). Hashimoto thyroiditis may exist before or it can develop after the diagnosis of AN. Although patients with Hashimoto thyroiditis generally present at the euthyroid state, Hashimoto thyroiditis either by causing hyper- or hypothyroidism could complicate the management of EDs.

Hypothyroidism caused by Hashimoto thyroiditis may complicate the management of AN by increasing the susceptibility to gain weight, thus inducing the AN patient to eat less. Hashimoto thyroiditis could also disrupt the management of AN by causing hyperthyroidism. The literature contains many reports on hyperthyroidism, mostly due to Graves’ disease, worsening AN either by leading to weight loss itself or by increasing the psychological distress by causing hyperphagia, weight gain, and a fear of losing self-control over eating (10,11,12). In the clinical course of our patient, Hashimoto thyroiditis disrupted the management of AN first by causing hyperthyroidism and then by leading to hypothyroidism. In the presence of AN, treatment of Hashimoto thyroiditis is challenging as well. AN could negatively affect the treatment of Hashimoto thyroiditis by altering the body weight and the metabolic rate, as well as by causing drug non-compliance.

While following patients with AN, because malnutrition could mask the clinical manifestations, the diagnosis of hyper- or hypothyroidism might be overlooked. Therefore, further analysis is needed when the major complaint does not improve with sufficient weight gain or when the levels of fT_3_, fT_4_, and TSH are incompatible with the body weight and nutritional status. Initially, our case was thought to have hyperthyroidism as the autoantibodies were positive, TSH level was lower than expected, and fT_4_ level was at the upper limits. However, during follow-up and within a short period of time, TSH suppression disappeared, T_4_ level started to decrease, and findings consistent with hypothyroidism became evident. Bradycardia being resistant to weight gain but improving after levothyroxine treatment was probably related to hypothyroidism as well.

Differentiating between primary thyroid diseases and low T_3_ syndrome is important in the achievement of best treatment outcome. Levothyroxine treatment is not recommended in low T_3_ syndrome, because it may cause weight loss, which is often mediated by loss of muscle mass (13). Also, thyroid hormone therapy, by leading to a decrease in weight, could obstruct the evaluation of patient’s response to treatment or could lead to the deterioration of symptoms (14,15). On the other hand, levothyroxine treatment could be given when hypothyroidism due to a primary thyroid disease accompanies AN. This raises the question of when to start levothyroxine in the coexistence of these two conditions. For our patient, thyroid functions tests were monitored closely during his hospital admission, and levothyroxine was initiated two months after discharge when he had gained enough weight and had become metabolically stable. Another problem is that, depending on the beliefs and attitudes of patients with AN, they could either reject using levothyroxine as it may lead to hyperphagia and weight gain or misuse it to lose weight (7,16). In addition, weight fluctuations may complicate the dose adjustment of thyroid hormone therapy in these patients. Our patient`s thyroid hormone levels could only be stabilized after he began to take the recommended dosage of levothyroxine and preserved his target weight.

Review of the literature for the association of Hashimoto thyroiditis with EDs revealed only one recently published case report of a 16-year-old girl with a 4-year history of AN whose TSH levels had increased in response to weight gain and shown a decrease with weight loss. This unexpected pattern of TSH and markedly elevated levels of antithyroperoxidase antibodies suggested Hashimoto thyroiditis (17). The association between body weight and TSH levels observed in this case report was also present in our patient. With weight restoration, an elevation in TSH levels occurred and levothyroxine treatment was started after TSH levels continued to rise with increasing weight similar to the case cited above. However, since levothyroxine had to be started in an earlier phase due to the hormonal status, the pattern of weight loss associated with decreases in TSH was not observed in our patient. Also, unlike the cited case, our patient was diagnosed with AN and Hashimoto thyroiditis at the same time. Moreover, initial tests of our patient being consistent with hyperthyroidism and misusage of levothyroxine created additional challenges during the follow-up.

Although Hashimoto thyroiditis is accepted as one of the most common autoimmune endocrine disorders, its effect on EDs seems to be lacking in the literature. This case of AN in conjunction with Hashimoto thyroiditis is informative as it highlights the challenges faced during the diagnosis and treatment of Hashimoto thyroiditis in the presence of AN and attracts attention to the effect of this coexistence on the patient.

## Ethics

Informed Consent: It was taken.

Peer-review: External peer-reviewed.

## Figures and Tables

**Table 1:**
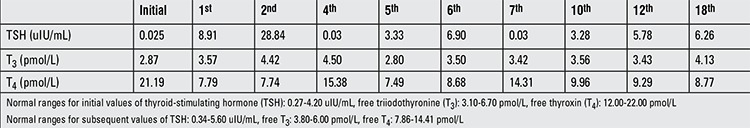
Thyroid function test results of the patient (initial values and subsequent monthly results)
